# Supplementary material for: Evidence for hierarchical representations of written and spoken words from an open-science human neuroimaging dataset
Source: Imaging Neurosci (Camb). 2026 Oct 15;4:IMAG.a.1388. doi: 10.1162/IMAG.a.1388 (PMC13673309; doi:10.1162/IMAG.a.1388)
Supplement: Supplementary file 1 [file IMAG.a.1388_supp.pdf]

SUPPLEMENTAL INFORMATION for *"Evidence for hierarchical representations of written and spoken words from an open-science human neuroimaging dataset"*

Suneel Banerjee, Kimberly Jin, Plamen P. Nikolov, Philip Cho, Vishnu R. Pendri, Lillian Chang, Srikanth R. Damera, Maximilian Riesenhuber

1. Calculation of bigram and syllable frequencies

Filtering of SUBTLEX-NL database

We sourced word frequencies from the "Zipf" column provided in the SUBTLEX-NL lexical database. Before calculating bigram and syllable frequencies for the Dutch language, we cleaned and filtered the SUBTLEX-NL database to remove artifacts left over from text mining of the corpus. These include onomatopoetic words, strings of special characters, misspelled words which do not meaningfully contribute to an estimation of the frequencies of letter combinations and syllables found in everyday Dutch usage.

- **Alphabetic Characters**
  - Retained words composed of standard Dutch letters (including diacritics).
  - Excluded entries containing digits, multiple punctuation marks, or symbols in non-standard positions.
- **Word Length Threshold**
  - Discarded words exceeding a predetermined character limit (25 characters, excluding hyphens). Excessively long strings are likely to be multi-word phrases or processing artifacts rather than individual Dutch words.
  - Discarded single-letter words (e.g., "u") as these could not be parsed into bigrams.
- **Handling 'SPEC' Entries**
  - The tag "SPEC" indicates special categories (e.g., certain proper nouns or abbreviations) in the SUBTLEX-NL database.
  - Retained "SPEC" words only if they were strictly alphabetic. Entries mixing letters with non-standard symbols were excluded.
- **Apostrophe Pattern**
  - Excluded words that begin with a single letter followed by an apostrophe (e.g., "a'trom"), as such patterns are non-standard in Dutch orthography.
- **Repeated Characters and Consecutive Letters**
  - Removed entries composed of a single, repeated letter (e.g., "aaaaa"), as those were likely errors or placeholders rather than genuine words.
  - Excluded any word containing three identical letters in a row (e.g., "aaangepaste").
  - Triple letters are exceedingly rare in standard Dutch and often indicate typos or informal elongations.
- **Text entries incompatible with syllabification algorithm**

- The syllabification algorithm published in Lathouwers et al., 2025 and utilized here to segment Dutch words into syllables is unable to process certain characters (accent marks, apostrophes, umlauts, etc.). Thus, 7605 words in the SUBTLEX-NL database were removed (in addition to the entries excluded by the criteria above) prior to calculating syllable frequency. However, only 4 unique words used in the MOUS language tasks could not be modeled as they contained apostrophes.

- **Number of entries failing each criterion (independently):**

  1) Invalid word pattern: 153807

  2) Exceeding length threshold: 3055

  3) Solely punctuation: 37

  4) SPEC with non-alphabetic chars: 106539

  5) Single-letter + apostrophe: 506

  6) Entirely repeated characters: 173

  7) Triple consecutive letters: 953

  8) Unable to be syllabified: 7605

Of 437503 entries in the database, a total of 159442 were dropped prior to calculating bigram frequencies, and a total of 167047 were dropped prior to calculating syllable frequencies. 29 words used in the MOUS language tasks did not have corresponding entries in the SUBTLEX-NL database; thus the onsets of these words were excluded from all lexical and sublexical Statistical Parametric Mapping (SPM) models.

2. Additional Lexical Contrasts

We also explored potential *positive* correlations between word frequency and brain activity during reading and speech listening.

T > 5.39
k > 20 voxels

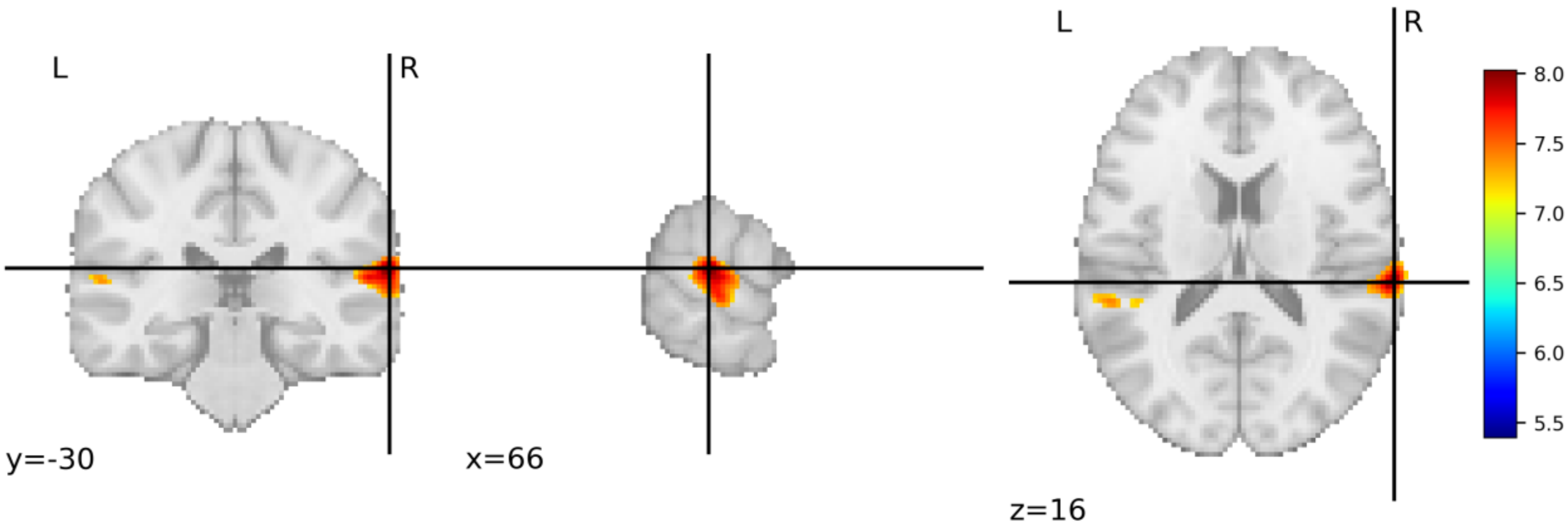

X	Y	Z	Region	Size (voxels)	Cluster Peak T
66	-30	16	Superior Temporal Gyrus, posterior division	495	8.02
-56	-38	16	Planum Temporale	95	6.19

T > 4.71
k > 20 voxels

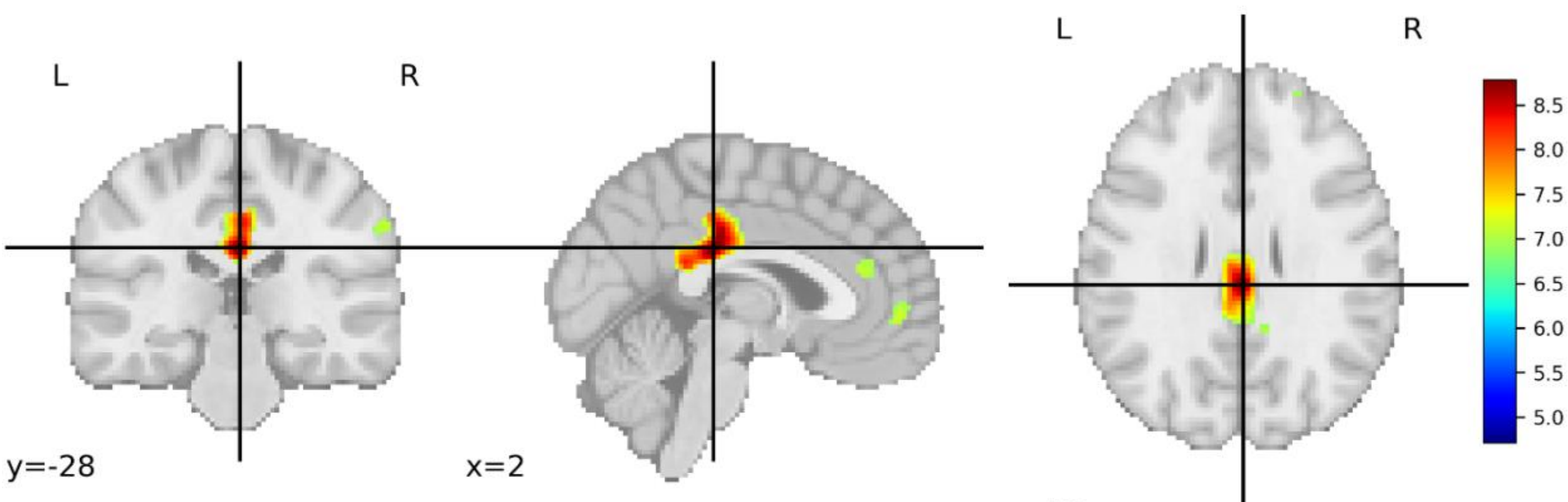

X	Y	Z	Region	Size (voxels)	Cluster Peak T
2	-28	28	Cingulate Gyrus, posterior division	716	8.78
8	44	-4	Paracingulate Gyrus	135	5.77
42	22	42	Middle Frontal Gyrus	51	5.77
4	36	18	Cingulate Gyrus, anterior division	32	5.25
64	-28	38	Supramarginal Gyrus, anterior division	23	5.21

Figure S1. Positive parametric effects of Zipf word frequency during reading (visual, top) and speech listening (auditory, bottom). Second-level SPM{T} maps (one-sample *t*-tests; reading N = 99, listening N = 101) for the Zipf word frequency parametric modulator, tested with a positive contrast weight (greater BOLD for higher-frequency words); $p < 0.05$ FWE-corrected, $k \geq 20$ voxels. Color bar indicates T (neurological convention, L = L).

3. Additional Sublexical Contrasts

In addition to the negative correlation with minimum bigram/syllable frequencies described in the main text, we also explored positive and negative correlations between the maximum and mean bigram and syllable frequencies found in a word and brain activity during reading and speech listening. No significant correlations between brain activity and maximum syllable frequency were observed.

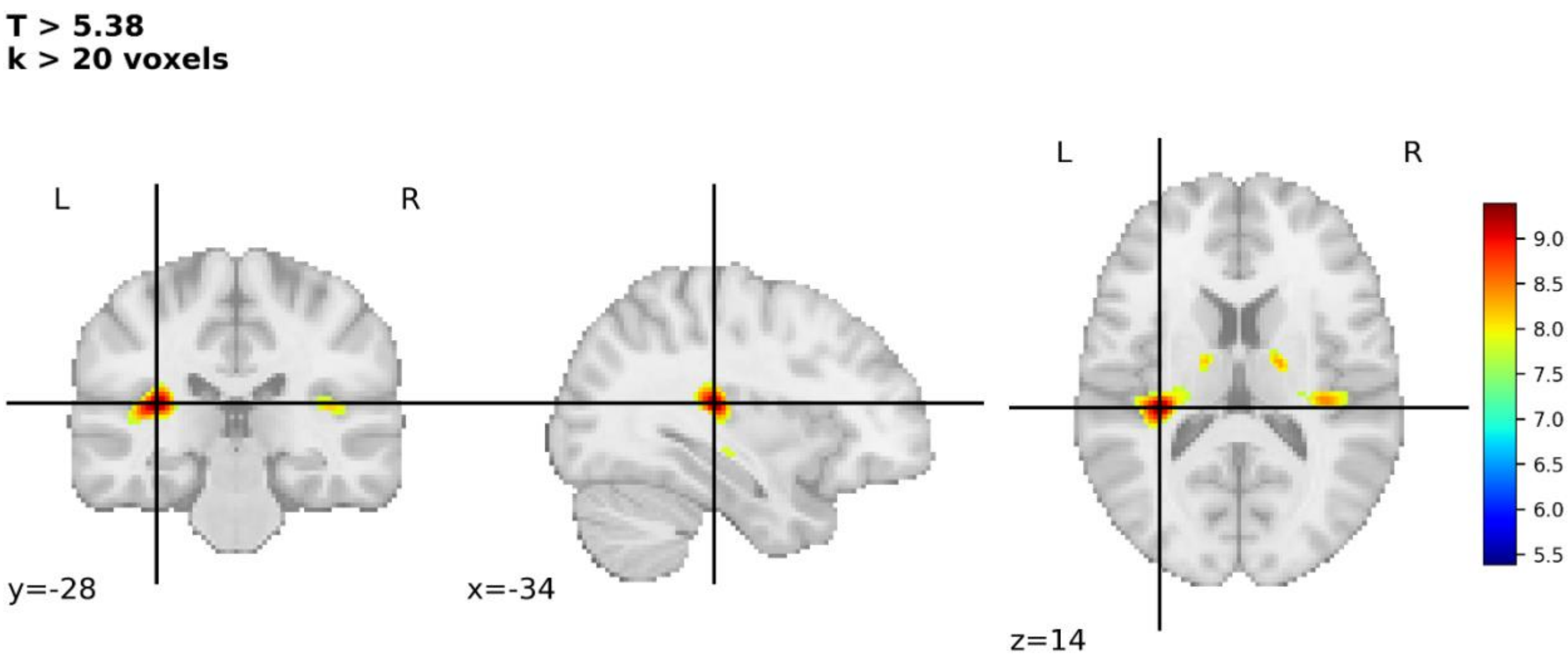

X	Y	Z	Region	Size (voxels)	Cluster Peak T
-34	-28	14	Heschl's Gyrus (includes H1 and H2)	558	9.38
50	-16	8	Heschl's Gyrus (includes H1 and H2)	355	7.91
-22	10	20	Deep White Matter / CSF	43	6.32
22	2	22	Deep White Matter / CSF	37	7.05
-32	-20	-8	Insular Cortex	22	6.38
26	2	-10	Deep White Matter / CSF	22	6.24

Figure S2. Whole-brain parametric correlation of BOLD with minimum bigram frequency during spoken-word processing. Second-level SPM{T} map (one-sample *t*-test, N = 101) for the minimum bigram frequency parametric modulator in the auditory runs, thresholded at T = 5.38 ($p < 0.05$ FWE-corrected), $k \geq 20$ voxels. Sections centered on the left Heschl's gyrus peak (crosshair; neurological convention, L = L); color bar indicates T. Effects appear bilaterally in Heschl's gyrus, left insula, and bilateral deep white-matter/CSF-adjacent regions.

T > 4.71
k > 20 voxels

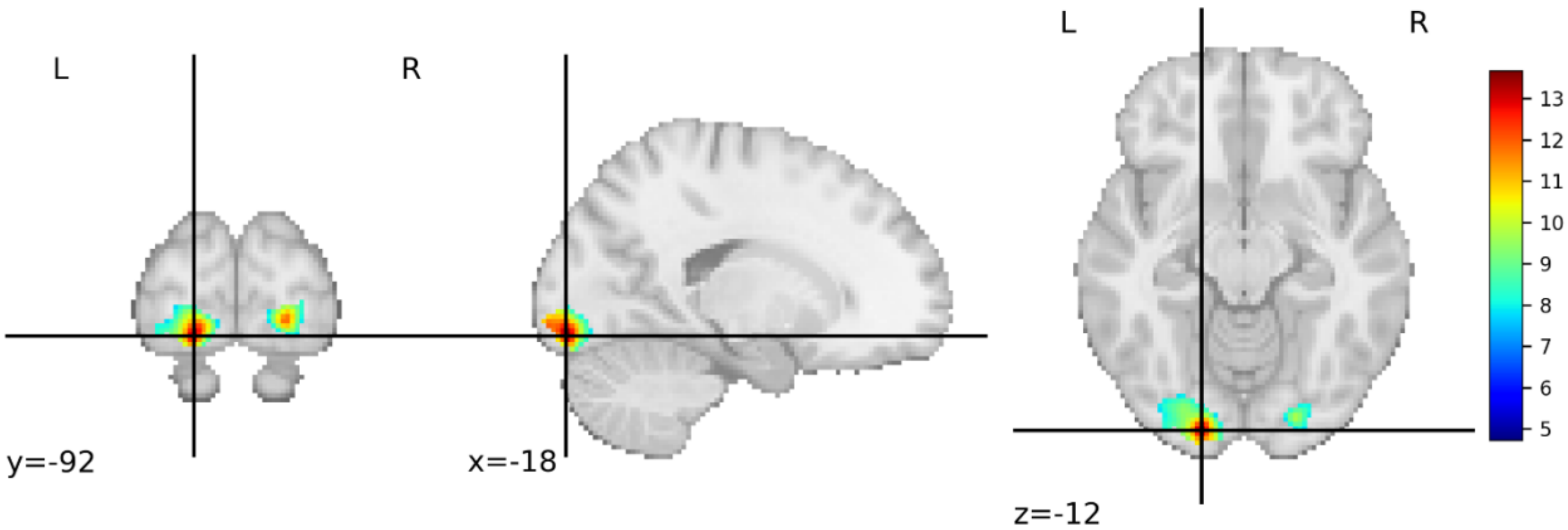

X	Y	Z	Region	Size (voxels)	Cluster Peak T
-18	-92	-12	Occipital Pole	683	13.67
22	-92	-6	Occipital Pole	322	10.82

T > 4.70
k > 20 voxels

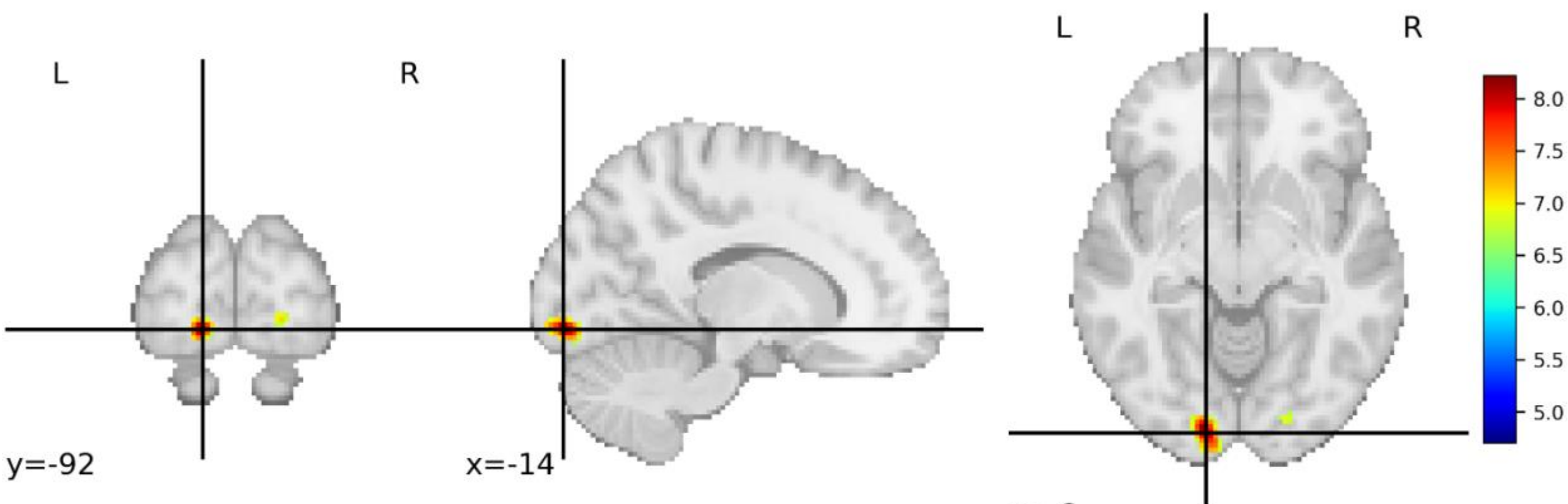

X	Y	Z	Region	Size (voxels)	Cluster Peak T
-14	-92	-8	Occipital Pole	163	8.22
20	-90	-4	Occipital Pole	44	5.81

Figure S3. Parametric effects of positive maximum (top) and mean (bottom) bigram frequency during reading. Second-level SPM{T} maps (one-sample *t*-test, N = 99) for the maximum and mean bigram frequency parametric modulators in the visual runs; pFWE < 0.05, k ≥ 20 voxels. Color bar indicates T (neurological convention, L = L).

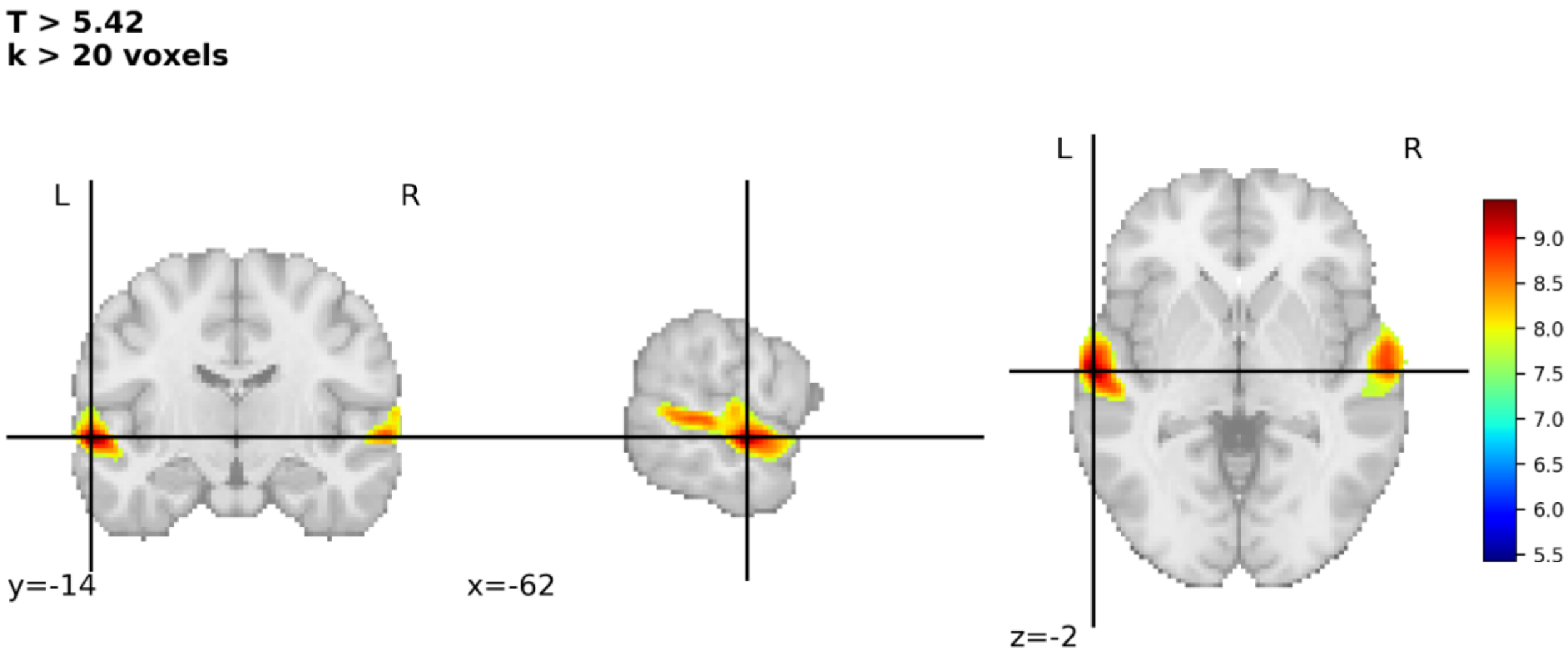

X	Y	Z	Region	Size (voxels)	Cluster Peak T
-62	-14	-2	Superior Temporal Gyrus, posterior division	1354	9.42
64	-4	-6	Superior Temporal Gyrus, anterior division	758	8.89
-48	22	20	Inferior Frontal Gyrus, pars opercularis	58	6.18
-44	-46	-16	Inferior Temporal Gyrus, temporooccipital part	25	5.88
-40	34	-10	Frontal Orbital Cortex	25	6.44
-48	34	4	Inferior Frontal Gyrus, pars triangularis	21	5.89

Figure S4. Parametric effects of negative mean syllable frequency of words during speech listening. Second-level SPM{T} maps (one-sample *t*-test, N = 101) for the log-transformed mean syllable frequency parametric modulators in the auditory runs; pFWE < 0.05, k ≥ 20 voxels. Color bar indicates T (neurological convention, L = L).

Unthresholded Contrast Images

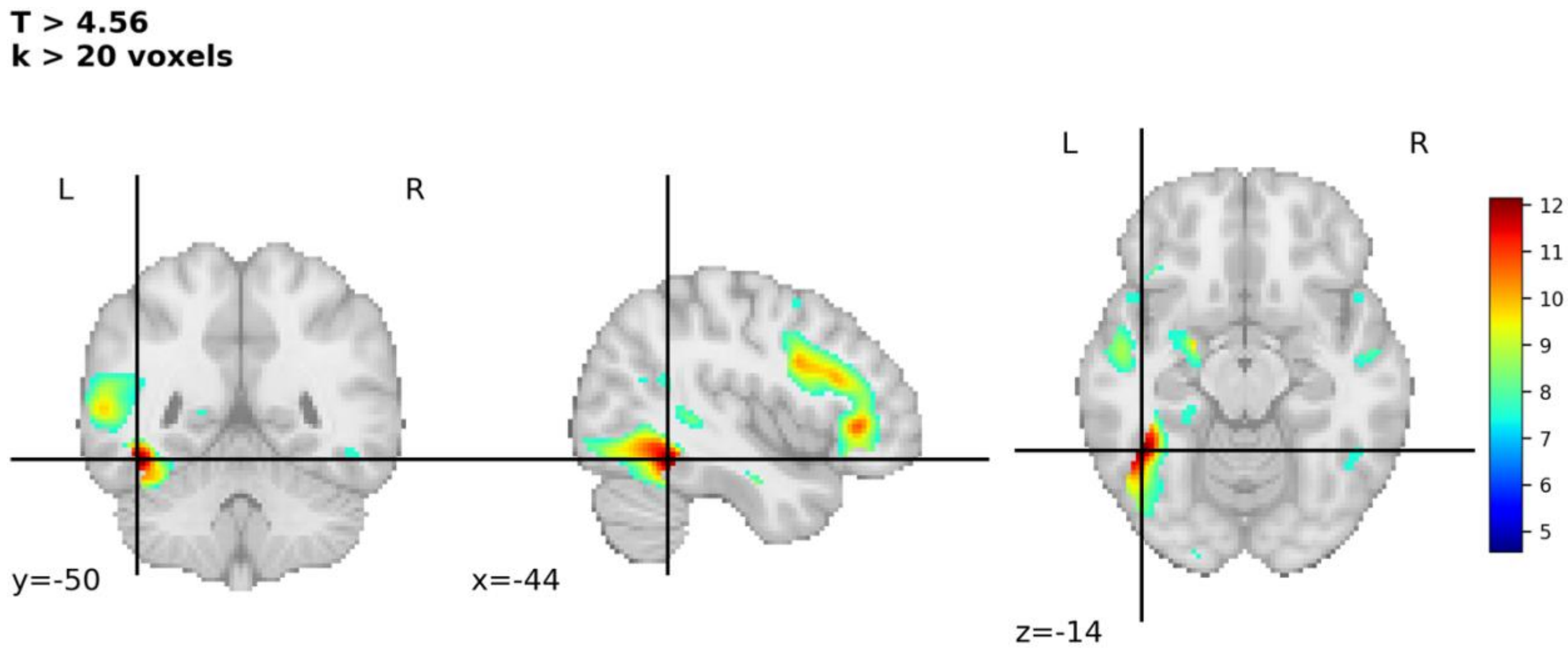

X	Y	Z	Region	Size (voxels)	Cluster Peak T
-48	30	-2	Inferior Frontal Gyrus, pars triangularis	2484	10.60
-56	-12	-6	Superior Temporal Gyrus, posterior division	1794	9.36
-44	-50	-14	Inferior Temporal Gyrus, temporooccipital part	1574	12.14
48	20	26	Inferior Frontal Gyrus, pars opercularis	414	6.13
42	-84	-6	Lateral Occipital Cortex, inferior division	193	6.12
-22	-4	-14	Deep White Matter / CSF	115	8.11
58	-6	-10	Superior Temporal Gyrus, anterior division	107	5.73
-4	14	58	Superior Frontal Gyrus	77	5.40
-40	4	50	Middle Frontal Gyrus	52	5.36
50	-30	2	Superior Temporal Gyrus, posterior division	52	5.38
32	-36	-22	Temporal Fusiform Cortex, posterior division	46	5.59
38	34	-12	Frontal Pole	44	6.02
16	-76	-40	Deep White Matter / CSF	40	5.54
-12	56	30	Frontal Pole	33	5.31
-6	42	-20	Frontal Medial Cortex	33	5.52
48	-50	-12	Inferior Temporal Gyrus, temporooccipital part	26	4.96

Figure S5a. Unthresholded parametric effects of word frequency during reading. pFWE < 1, k ≥ 20 voxels. This contrast corresponds to that applied in Figures 1 & 3.

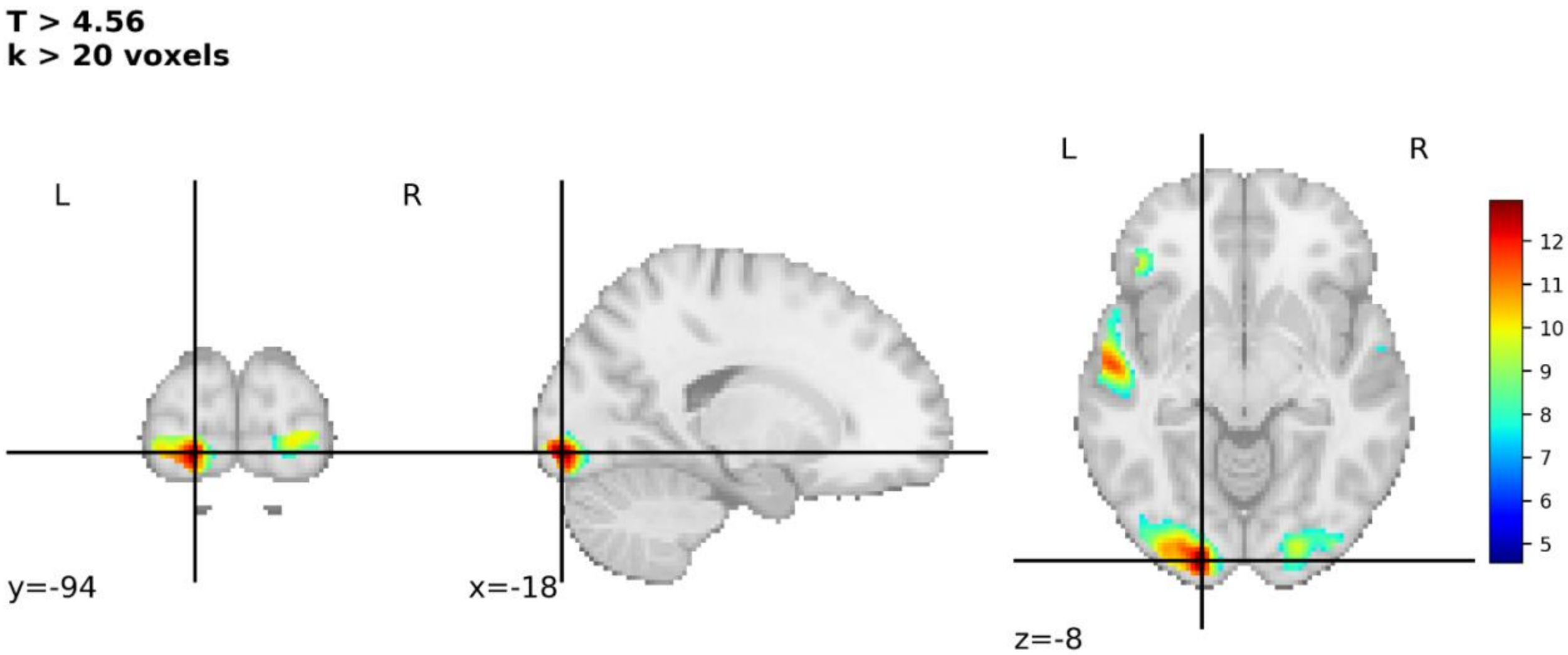

X	Y	Z	Region	Size (voxels)	Cluster Peak T
-58	-10	-8	Superior Temporal Gyrus, posterior division	1887	10.77
-18	-94	-8	Occipital Pole	1273	12.93
-46	32	-2	Inferior Frontal Gyrus, pars triangularis	778	9.25
24	-96	-2	Occipital Pole	435	8.83
-2	44	-20	Frontal Medial Cortex	121	7.04
-10	58	34	Frontal Pole	86	6.18
54	-8	-14	Superior Temporal Gyrus, posterior division	29	6.01

Figure S5b. Unthresholded parametric effects of bigram frequency during reading. pFWE < 1, k ≥ 20 voxels. This contrast corresponds to those applied in Figures 2 & 3.

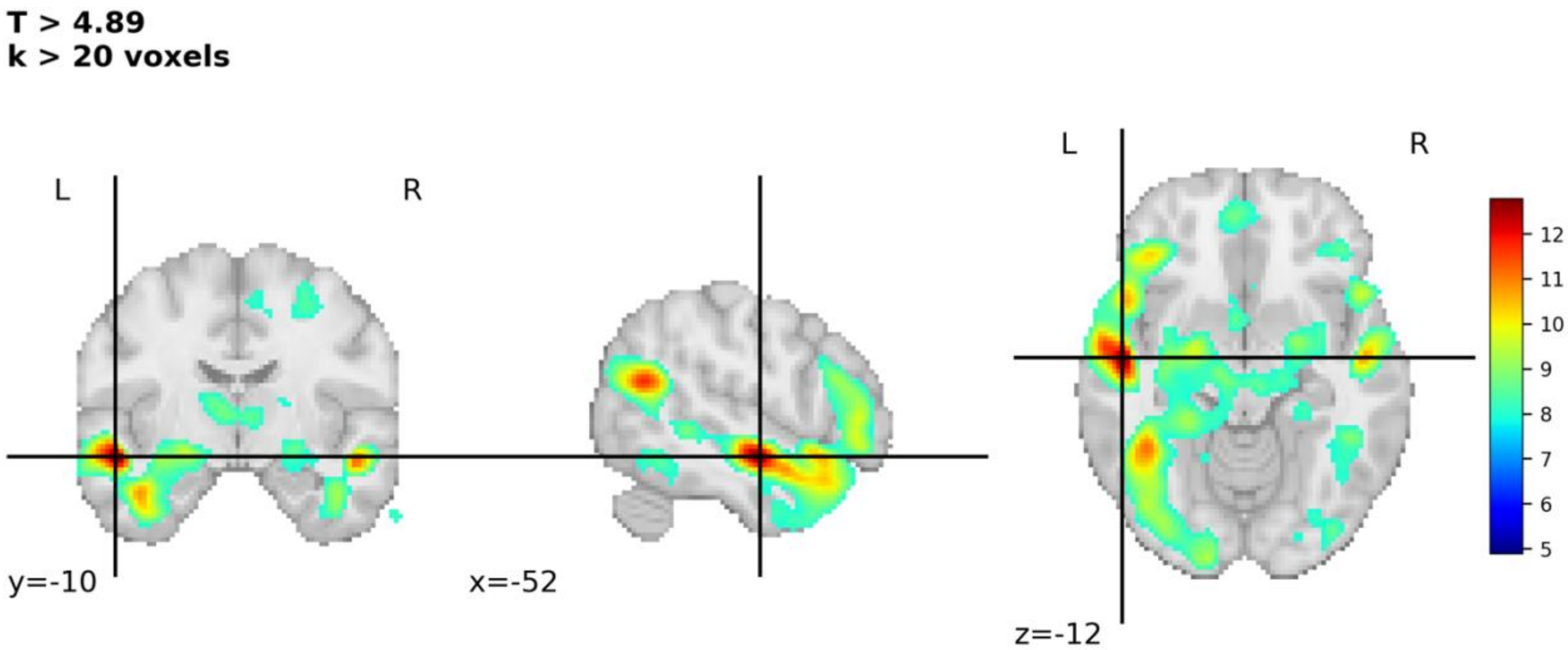

X	Y	Z	Region	Size (voxels)	Cluster Peak T
-52	-10	-12	Superior Temporal Gyrus, posterior division	21728	12.77
50	-10	-14	Superior Temporal Gyrus, posterior division	3388	9.99
10	-2	52	Juxtapositional Lobule Cortex (formerly Suppleme	695	6.82
-8	56	36	Frontal Pole	606	8.08
0	-56	-38	Deep White Matter / CSF	518	6.54
42	-20	52	Postcentral Gyrus	321	6.31
12	-76	-40	Deep White Matter / CSF	268	6.19
44	0	16	Central Opercular Cortex	113	6.56
42	36	-12	Frontal Pole	110	6.08
-22	-32	-40	Deep White Matter / CSF	99	5.42
-6	-74	-36	Deep White Matter / CSF	58	5.38
50	-64	20	Lateral Occipital Cortex, superior division	54	5.44
20	-36	-40	Deep White Matter / CSF	53	5.80
28	26	2	Insular Cortex	33	5.55
0	-36	-58	Deep White Matter / CSF	26	5.36
48	-22	22	Parietal Opercular Cortex	20	5.24

Figure S6a. Unthresholded parametric effects of word frequency during speech listening. pFWE < 1, k ≥ 20 voxels. This contrast corresponds to those applied in Figures 4 & 6.

T > 4.89
k > 20 voxels

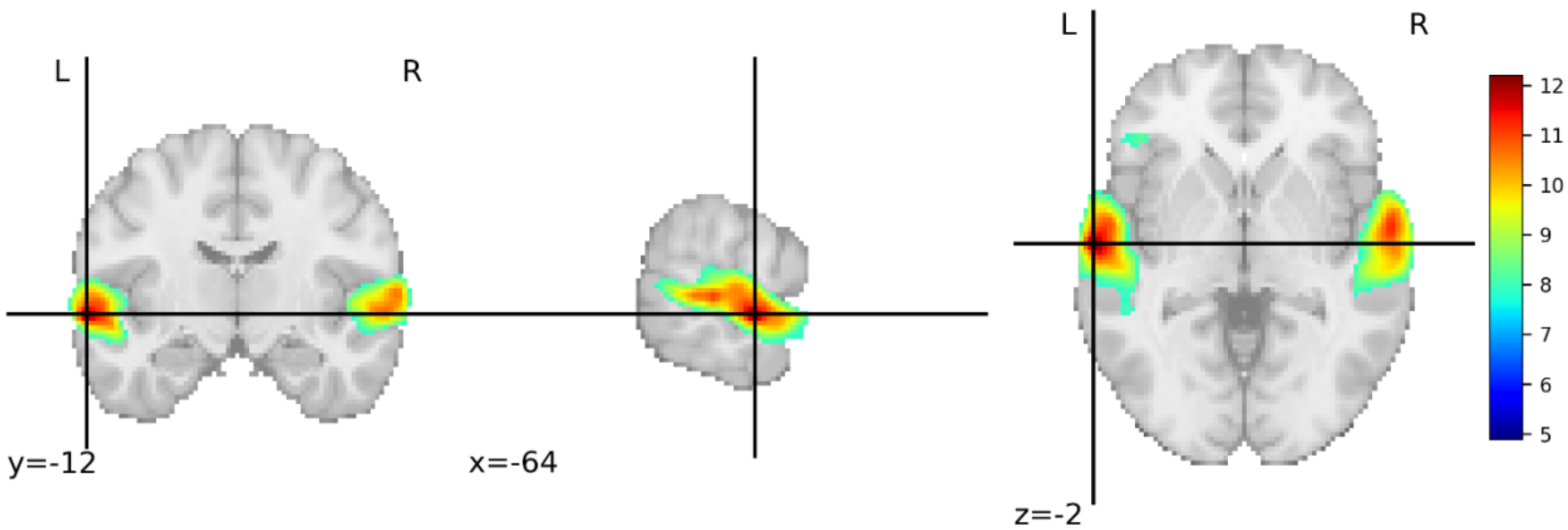

X	Y	Z	Region	Size (voxels)	Cluster Peak T
-64	-12	-2	Superior Temporal Gyrus, posterior division	3455	12.19
64	-4	-4	Superior Temporal Gyrus, anterior division	2484	11.25
-50	32	4	Inferior Frontal Gyrus, pars triangularis	162	5.95
-42	-42	-18	Temporal Fusiform Cortex, posterior division	81	6.12
-46	16	26	Inferior Frontal Gyrus, pars opercularis	50	5.43

Figure S6b. Unthresholded parametric effects of syllable frequency during speech listening. pFWE < 1, k ≥ 20 voxels. These contrasts correspond to those applied in Figures 5 & 6.

4. Correlations between lexical and sublexical features

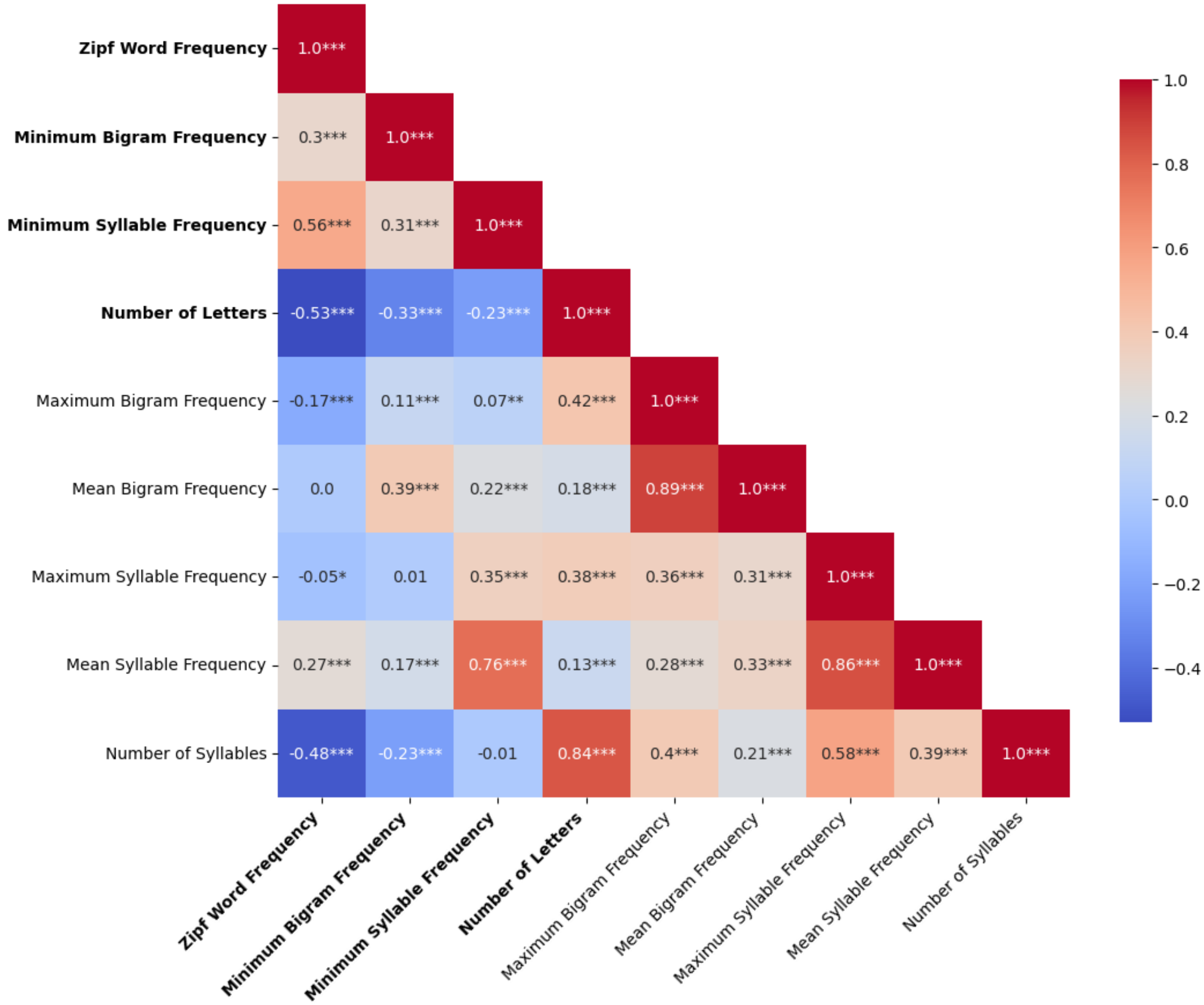

Figure S7. Correlations between lexical and sublexical features. The matrix displays pairwise Pearson correlation coefficients among nine word-level parameters, including word frequency, log-transformed bigram and syllable frequency measures, and structural characteristics (letter and syllable counts) for all modeled words (n = 1910) that appeared in the MOUS language tasks. Asterisks denote statistical significance of each correlation coefficient (p < .001: ****)*. Bolded axis labels indicate the four features that were ultimately selected as parametric modulators in the fMRI general linear models. Not shown above is spoken word duration in seconds, which was included as a parametric modulator in the auditory word frequency correlation contrast, but is not a constant lexical characteristic. While log-transformed maximum and mean frequencies for syllables and bigrams were initially considered as candidate parametric regressors for fMRI analysis they were excluded due to lack of explanatory power (see Figures S3 & S4).

5. Analyses with additional regressors

Our analyses in the paper focused on correlations with lexical and sublexical frequency alone. This not only allows comparison to the prior literature that used the frequency correlation technique (Bruno et al., 2008; Carreiras et al., 2006; Chee et al., 2003; Graves et al., 2010; Kronbichler et al., 2004), but the advantage of these single correlations is their straightforward interpretability, testing the hypotheses that the activation of a lexical representation consisting of neurons holistically tuned to words (see (Glezer et al., 2009) correlates inversely with word frequency, and the activation of a sublexical representation consisting of neurons tuned to sublexical units correlates inversely with the frequency of the most infrequent sublexical unit.

To explore the questions what activations in response to bigram/syllable frequency are not shared by written/auditory word frequency, and vice versa, we have also performed additional analyses that included sublexical and lexical frequency measures in the same general linear model (GLM) for each sensory modality. In each sensory modality, we explore the effect of one parameter while treating the other as a parameter of no interest. This allows us to examine the effect of word frequency above and beyond that which can be explained by bigram/syllable frequency and vice-versa.

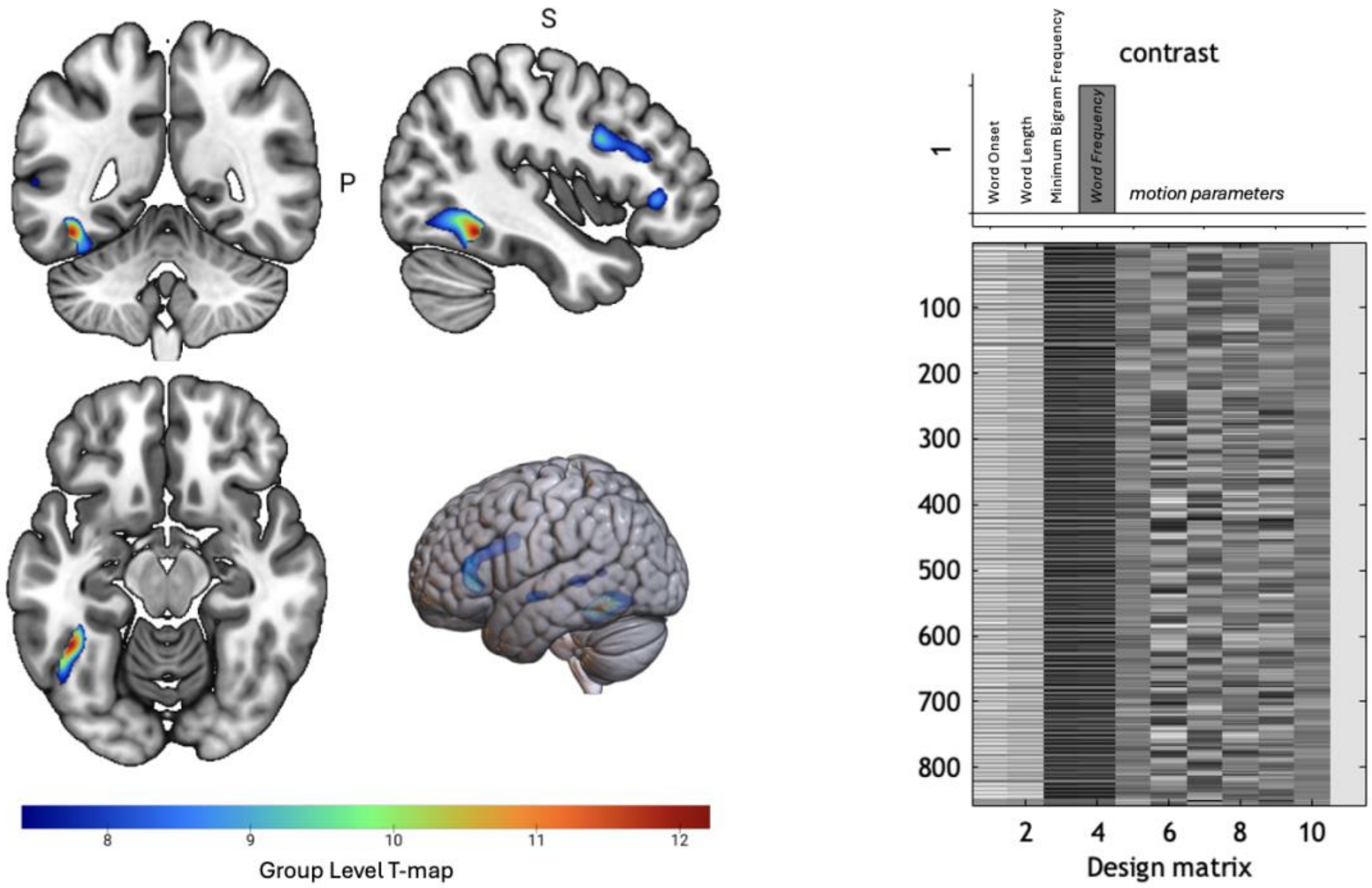

Cluster Peak (MNI)	Region (Brodmann Area)	Extent (voxels)	Peak T-value
-44, -50, -14	*Left fusiform gyrus (37)*	409	12.21*
-52, 34, 2	*Left inferior frontal gyrus pars triangularis (45)*	678	10.12
-56, -8, -8	*Left superior temporal gyrus (22)*	58	8.95
-60, -34, 4	*Left middle temporal gyrus (21)*	121	8.49
-60, -52, 8	*Left angular gyrus (11)*	20	7.91

Figure S8. The effect of word frequency when including bigram frequency as a regressor of no interest in a combined model of responses to written words. Top left: Rendering of group-level effect of word frequency (controlling for word length and minimum bigram frequency). Top right: Example SPM12 contrast vector and design matrix showing all parametric regressors included in a general linear model for a single subject. Bottom: Table listing all significant clusters (pFWE < $1e^{-6}$, k ≥ 20 voxels.).

We find significant clusters associated with word frequency in the VWFA region during reading, even when including minimum bigram frequency as a regressor of no interest. The strength of our peak cluster (asterisk), located in the VWFA, increases by 0.07 (relative to Table 1) when including bigram frequency as a regressor of no interest. The occipital cluster we previously observed not only in the sublexical (Table 2) but also in the word frequency analysis (Table 1) no longer shows significant correlation with word frequency when including minimum bigram frequency as a regressor of no interest. These results reinforce that the VWFA is selectively sensitive to whole-word representations beyond simple letter co-occurrence patterns, and that the posterior cluster is selective at the sublexical but not the lexical level.

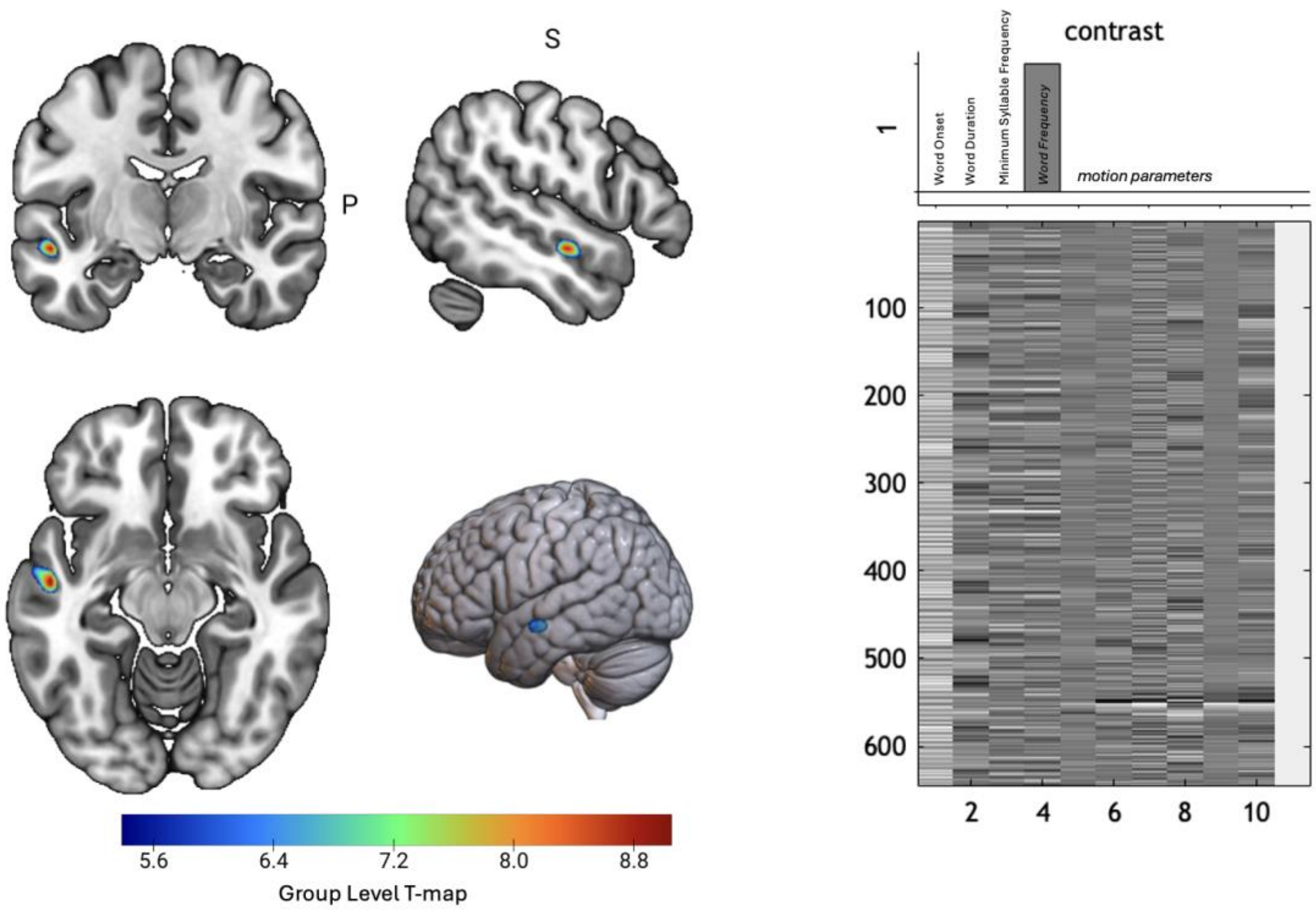

Coordinates	Region (Brodmann Area)	Extent	Peak T- value
-52, -10, -12	*Left Superior Temporal Gyrus (22)*	80	9.05

Figure S9. The effect of word frequency when including syllable frequency as a regressor of no interest in a combined model of responses to auditorily presented words. Top left: Rendering of group-level effect of word frequency (controlling for word duration and minimum syllable frequency). Top right: Example SPM12 contrast vector and design matrix showing all parametric regressors included in a general linear model for a single subject. Bottom: Table listing all significant clusters (pFWE < $1e^{-6}$, k ≥ 20 voxels.

For speech listening (Fig. S9, above), we find only one surviving cluster in in the superior temporal gyrus that is associated with word frequency when including syllable frequency as a regressor of no interest. The location of this cluster corresponds to that reported as the AWFA in the main manuscript. Thus, even when controlling for syllable frequency, we still observe that the activity of the AWFA is inversely correlated with the frequency of spoken words, providing further support for the AWFA's hypothesized role as an auditory lexicon.

In contrast to the lexical frequency correlation results, we do not find noteworthy significant correlation clusters associated with sublexical frequency when including lexical frequency in the same model.
